# Is hypoxia good or bad? Role of oxygen levels in maize kernel development

**DOI:** 10.1093/plphys/kiad146

**Published:** 2023-03-05

**Authors:** Maria-Angelica Sanclemente

**Affiliations:** Assistant Features Editor, Plant Physiology, American Society of Plant Biologists, USA; Plant Stress Resilience, Utrecht University, Utrecht 3584 CH, Netherlands

Cellular functions of molecular oxygen are diverse. The most prominent role is as a substrate for oxidative phosphorylation and ATP production in the mitochondria. Low oxygen (hypoxia) can thus be detrimental for most plant tissues if levels drop below the threshold needed to sustain respiratory demands (Sasidharan et al. 2021). For instance, oxygen deficiency can quickly shift from aerobic production of 36 ATP to 2 ATP produced during glycolysis and fermentation (van Dongen and Licausi 2015).

Despite these negative effects, hypoxia is common in nonphotosynthetic tissues with high metabolic demands and high cell density, such as meristems and seeds (Borisjuk and Rolletschek 2009; Weits et al. 2019). However, in these organs, oxygen limitation is not detrimental, but instead is central to their development (Kelliher and Walbot 2012, 2014). For example, maize (*Zea mays*) kernels normally grow with a low-oxygen, high-sugar environment inside their endosperm (Rolletschek et al. 2005).

Early work by Rolletschek et al. (2005) has shown that steep oxygen gradients occur inside the maize endosperm, while levels are 10-fold higher in the embryo. The work indicates that oxygen gradients influence the fate of resource partitioning, local ATP concentration, and metabolite distribution. Moreover, transcriptional modifications are also evident in hypoxic niches (Koch et al. 2000; Gayral et al. 2017). All of these components favor starch accumulation in the hypoxic endosperm and lipid accumulation in the more oxygenated embryo. Questions remain about the mechanisms and other features driving the formation of oxygen gradients inside maize kernels, their effect on gene expression, signaling, and metabolism, and the temporal dynamics during seed development.

All of these questions have been addressed by Langer et al. (2023). In this issue of *Plant Physiology*, the authors found that development of localized hypoxic environments inside maize kernels is not a result of domestication but a conserved feature observed in modern maize and wild relatives. All the genotypes tested showed a steep reduction in oxygen levels starting at the apical portion of the kernel [immediately below the surface (100 to 400 µm)] and extending throughout the endosperm. Levels increased sharply at the basal transfer layer (lower endosperm) which remains oxygenated (Fig. 1A). The profile of oxygen levels paralleled that of starch and water distribution during seed filling. Starch accumulation in the upper-mid endosperm displaced water towards more oxygenated regions in the transfer layer and embryo (Fig. 1B). These oxygen and starch/water gradients suggested a potential role for the chalazal pericarp at the basal end of the kernel in the maintenance of oxygenated locales. Using X-ray µ-CT, the authors found a porous layer in the basal endosperm that extends towards the embryo (Fig. 1C). This identified void network has high diffusivity and can provide more than 40% of the oxygen that enters the endosperm and that is available to sustain embryonic respiratory demands.

**Figure 1.**
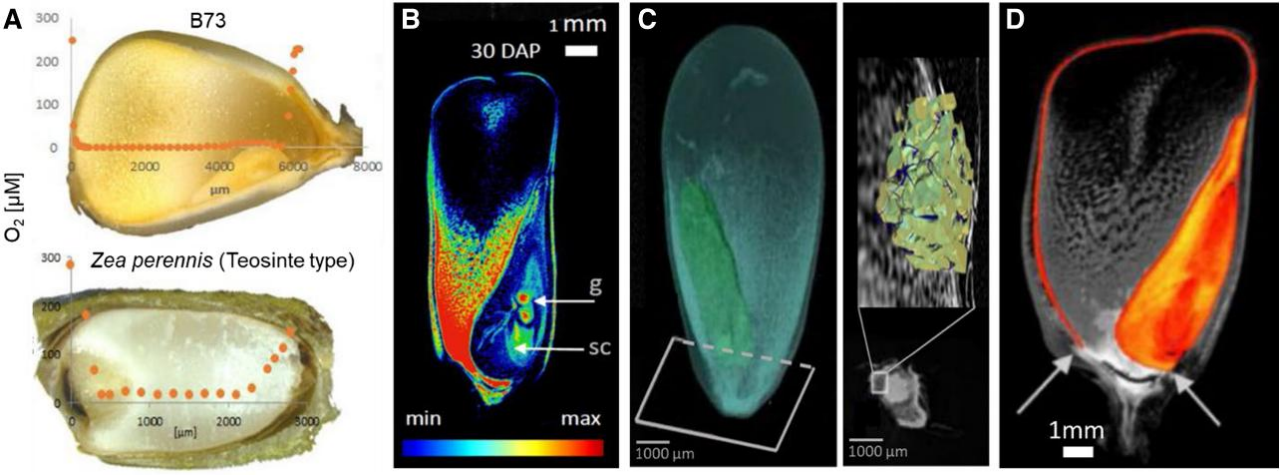
Topological characteristics of different components of mature maize kernels. **A)** Oxygen concentration profile of modern maize (B73) and wild-relative teosinte. **B)** Distribution of water signals (color-coded) in mature kernels measured by MRI. Abbreviations: DAP, days after germination; g, germ; sc, scutellum. **C)** 3D image of a B73 kernel with the segmented porous layer (blue) and embryo (green). Image was acquired using μ-CT imaging analysis with a pixel resolution of 5 μm. A subplot of a horizontal cross-section through the CT scan site at the base of the kernel shows the porous pericarp with a well-connected pore network (blue) through tissue (yellow). **D)** Kernel lipid distribution as detected by MRI shows the presence of a lipidous layer at the endosperm surface and embryo. Arrows indicate the lack of lipidous layers in the chalazal endosperm region. Adapted from Langer et al. (2023), Figs. 1B, 2, 3A and 3B, and Fig. 4H.

The lack of porous layers in the endosperm and outer pericarp was consistent with a higher resistance to gas diffusion and lower oxygen levels. These results, together with the observation that empty kernel sections in *sugars will eventually be exported transporter 4c* (*sweet4C*) mutants remained oxygenated, indicated the presence of a diffusion barrier at the endosperm level rather than at the nonphotosynthetic pericarp as has been previously suggested (Rolletschek et al. 2005; Borisjuk and Rolletschek 2009). The enhanced diffusion resistance of the endosperm and internal oxygen consumption can result in steep gradients in this tissue. Moreover, the presence of a lipidous layer in the aleurone and cutin/wax layers that encapsulate the endosperm (Fig. 1D) also contributes to the restricted permeability of the endosperm during kernel development. Notably, these lipidous layers are absent in the chalazal section of the endosperm, potentially increasing gas diffusion in this region.

The authors tested the importance of oxygen levels on kernel development by manipulating the oxygen supply in intact seeds. Treatments with low, control, and high levels of oxygen revealed regulatory networks and metabolic components associated with hypoxia acclimation of maize kernels. Reciprocal gene responses to oxygen availability were associated with mitochondrial functions and diverse signals (i.e. auxin). These shifts were supported by adjustment of metabolic pathways related to energy, stress, and respiration. Notably, results here also showed that hypoxia slows down kernel development, while high oxygen accelerates development and changes resource partitioning.

The work of Langer et al. (2023) is exciting as it uncovered structural features of maize kernels and solves long standing questions about formation of endogenous oxygen gradients in this organ. The research also raises questions regarding the formation of porous layers on the chalazal region and the possibility of manipulating internal oxygen availability through the void network and the lipidous layers on the outer endosperm. Could kernel development be accelerated by increasing pore density or distribution? And could this approach also be used to manipulate resource allocation to the embryo or endosperm?

More work will be needed to test these possibilities and the contributions of the lipid and cutin/waxin layers on gas diffusion. Modeling and sophisticated visualization techniques used by Langer et al (2023) also provide a framework to study other aspects of kernel development, such as water relations and source/sink dynamics.

## Data Availability

Data presented here is available in Langer et al (2003; https://doi.org/10.1093/plphys/kiad038.
